# Foreign Body Aspiration – A Potentially Life-Threatening Complication of Seizures

**DOI:** 10.7759/cureus.11349

**Published:** 2020-11-05

**Authors:** Dean Redant, Zeyn Mahomed, Craig Beringer

**Affiliations:** 1 Emergency Department, Chris Hani Baragwanath Academic Hospital, University of the Witwatersrand, Johannesburg, ZAF

**Keywords:** epileptic seizures, foreign body aspiration

## Abstract

In adults, foreign body aspiration is an uncommon clinical presentation. Aspiration can occur during a seizure and in the post-ictal period due to the loss of airway reflexes. Commonly aspirated contents include saliva, blood, or vomited gastric contents. Due to a common misconception that placing an object, such as a spoon, in a seizing person’s mouth prevents tongue-biting, a variety of unusual items may also potentially be aspirated. With an unclear history, relatively small, radiolucent objects are often misdiagnosed or missed entirely. Chest pain or unexplained hemoptysis may be the only symptoms to suggest aspiration. In this report, the authors present a case of a patient with an unusual foreign body aspiration.

## Introduction

Foreign body aspiration is common in children; however, it is rarely diagnosed in adults [1]. Aspiration of foreign bodies by adult patients usually occurs in the elderly or in patients with pre-existing neurological or psychiatric disorders [2-4]. In some cases, tracheobronchial foreign bodies are detected early due to an indicative history or immediate respiratory symptoms ranging from mild pain to asphyxiation and respiratory arrest [3-7]. Many cases are misdiagnosed or missed entirely, only to be diagnosed much later due to persistent recurrent hemoptysis or recurrent suppurative lung infections [4-7]. Any item of appropriate size can be aspirated, and various bizarre objects have been noted in the literature, ranging from garlic bulbs to cinnamon sticks and razor blades to voice prostheses, with the majority being located in the right lower lobe bronchus [4-7].

Seizures are commonly seen in the emergency department (ED), yet are poorly understood in many communities. Associated inaccurate beliefs and misconceptions regarding seizures can result in the inappropriate management of a person experiencing it [8]. The following case highlights the dangers of one such practice.

## Case presentation

**Figure 1:**
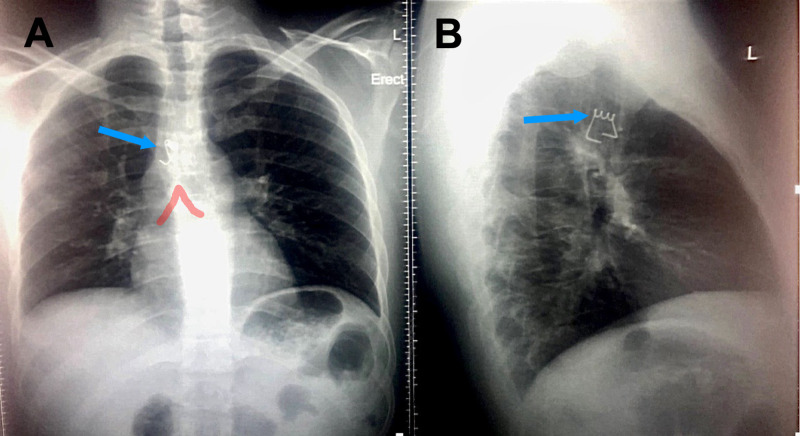
Chest radiograph: anterior-posterior (A) and lateral (B) views. (A) demonstrates the radio-opaque metal spring (blue arrow) just above the carina (red line) at the level of the right main bronchus. (B) confirms the location of the object (blue arrow) in the trachea, situated anterior to the esophagus.

A 29-year-old man presented to the ED with a one-day history of hemoptysis, central, pleuritic chest pain, and dyspnea. The patient is known with epilepsy and had experienced a generalized tonic-clonic seizure one day prior to the onset of symptoms. No history of aspiration or choking had been reported, and the patient recovered fully, returning to his normal baseline functional status after a brief post-ictal period. On examination, he had mildly decreased breath sounds on the right hemithorax. He was otherwise comfortable, with no signs of respiratory distress or stridor and normal oxygen saturations. A chest radiograph was ordered, and a metal spring could clearly be seen at the level of the carina. The spring was angled toward the right main bronchus on both anterior-posterior and lateral views (Figure 1).

**Figure 2:**
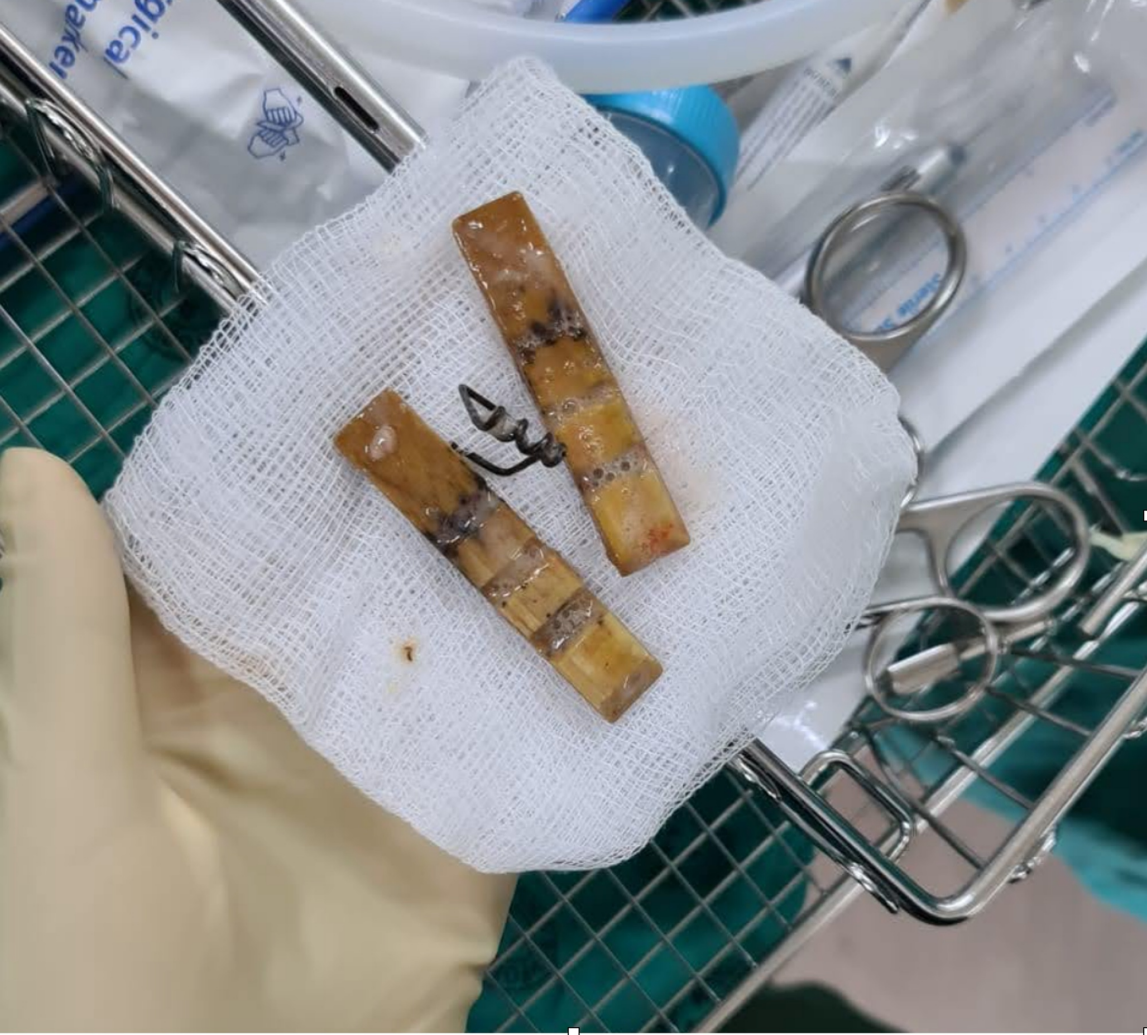
A wooden clothing peg removed from the lower trachea using rigid bronchoscopy.

Upon further interviewing, the patient’s wife informed the attending physician that a wooden clothing peg had been placed in the patient’s mouth during the seizure. The family members had done this with the belief that it would prevent him from biting his tongue. During the seizure activity, however, the displacement and aspiration of the peg into the patient’s airway had gone unnoticed by the family. Due to the anatomical location of the aspirated object, the patient was transferred to a cardiothoracic surgical unit. The patient was then taken to theater, where a wooden clothing peg was removed via rigid bronchoscopy (Figure 2). The patient recovered uneventfully and was discharged two days later.

## Discussion

Epilepsy is a common neurological condition seen in EDs worldwide with a higher prevalence and severity in lower socioeconomic countries [9]. Although the incidence of aspiration during seizures is poorly defined, limited research places it at under 1% [10]. An increased risk of aspiration has been associated with a higher frequency of seizures and baseline airway impairment [10]. Loss of consciousness during a generalized seizure as well as post-ictal confusion would make it difficult for the physician to obtaining a history suggestive of aspiration. In this report, the patient was not aware of any item being placed inside his mouth. Patients often have delayed presentations due to complications such as recurrent pneumonias, bronchostenosis, or intractable coughing [2,5,7,11-13].

The nature of tracheobronchial foreign bodies varies with age and geographic location. Bone fragments, vegetable matter, and metallic objects, particularly hairpins, are the most commonly aspirated objects [1,11-13]. The majority of organic matter, including small bones and wood, are not visible on standard chest radiographs; consequently, less than 30% of aspirated foreign bodies are visible on chest radiograph [11,13]. Nonspecific lung changes including heterogeneous opacities and atelectasis are more likely to be found on a chest radiograph. The chest radiograph can be expected to be normal in up to a quarter of patients requiring intervention [11].

While the myth of placing a spoon in a seizing person’s mouth may not appear to carry a risk of aspiration at face value, that risk arises if any item with a caliber smaller than the person’s trachea is inserted into their mouth. Placing a spoon in a seizing person’s mouth with the aim of preventing them from biting their tongue is still commonly believed and practiced in Sub-Saharan Africa [14]. As this case report illustrates, the item may not necessarily be a spoon or metallic and thus may not be visible on a routine chest radiograph.

When combining the poor specificity of history and examination, along with the paucity of visible foreign bodies on chest radiography, the diagnosis of foreign body aspiration can be difficult. As such, the diagnosis is likely to be missed without a high index of suspicion [3,5,13]. A patient with epilepsy adds an extra layer of complexity in that they cannot validate or deny one’s suspicion. A careful collateral history should be sought in order to ensure that no foreign body had been inserted in the patient’s mouth. It is also important to conduct an examination of the oral cavity to determine if there are any missing teeth or dentures, or signs of oral trauma from foreign body insertion [11,13]. If any suspicion remains for aspiration, further imaging should be ordered.

While rigid bronchoscopy is the gold standard for diagnosis and intervention, flexible bronchoscopy can be done in the ED and allows for immediate intervention [4-5,11-12]. A computed tomography scan of the chest is a valuable adjunct to aid diagnosis if immediate bronchoscopy is unavailable [11]. A missed diagnosis of an aspirated foreign body can lead to significant morbidity and potentially life-threatening complications.

## Conclusions

It is imperative to maintain a high index of suspicion for foreign body aspiration in all epileptic patients presenting with unexplained respiratory symptoms. Foreign bodies are frequently not seen on a chest radiograph. Detailed history taking and examination continue to serve as important tools in solving most diagnostic dilemmas. Furthermore, health-care professionals should actively educate their patients on the dangers of placing any item in the mouth of someone experiencing a seizure.
